# Predictors and Prognostic Significance of Appropriate Implantable Cardioverter-Defibrillator Therapy in Primary Prevention Patients with Ischemic Cardiomyopathy

**DOI:** 10.3390/jcm15031033

**Published:** 2026-01-28

**Authors:** Mateusz Kuśmierz, Jakub Mercik, Marek Śledziona, Barbara Brzezińska, Maria Łoboz-Rudnicka, Bogusława Ołpińska, Krzysztof Dudek, Rafał Wyderka, Krystyna Łoboz-Grudzień, Joanna Jaroch

**Affiliations:** 1 Department of Cardiology, Marciniak Lower Silesian Specialist Hospital-Emergency Medicine Center, 54-049 Wroclaw, Poland; 2 Faculty of Mechanical Engineering, Wroclaw University of Science and Technology, 50-370 Wroclaw, Poland; 3 Faculty of Medicine, Wroclaw University of Science and Technology, 50-370 Wroclaw, Poland

**Keywords:** implantable cardioverter-defibrillator, ischemic cardiomyopathy, appropriate therapy

## Abstract

**Background:** In the population of patients with ischemic cardiomyopathy (IC) and reduced left ventricular ejection fraction, the benefits of prophylactic implantable cardioverter-defibrillator (ICD) therapy are not uniform. Identifying predictors of ventricular arrhythmias to estimate the risk of appropriate therapy is crucial. **Methods:** Patients with IC and an ICD for primary prevention implanted between 2006 and 2019 were retrospectively analyzed for appropriate therapy (ATh). The primary objective was to assess predictors of ATh development. The secondary objective was to assess the impact of ATh on survival. **Results:** Overall, 260 patients (age 67.3 ± 9.4 years, 15.4% female) were analyzed with a follow-up of 4.47 ± 3.02 years. ATh occurred in 79 patients (30.4% of the study group). Independent risk factors for ATh were as follows: non-sustained ventricular tachyarrhythmias (nsVTs) detected before ICD implantation, extensive area of ischemic left ventricular damage on echocardiographic assessment, left ventricular end-diastolic dimension (LVEDd) ≥ 68 mm, history of coronary artery bypass grafting (CABG), and presence of chronic total occlusion (CTO). A multiparameter logit model was created to estimate the probability of ATh. Patients with a score ≥ 0.6 had more than a six-fold higher risk of developing ATh compared with patients with a score < 0.6. Patients after ATh had significantly lower survival compared to patients without intervention (HR 1.69, *p* = 0.008). **Conclusions:** Patients with the independent risk factors listed above are at higher risk for ATh. A multiparameter logit model based on these risk factors is effective in estimating the risk of ATh. The occurrence of ATh was associated with a significantly higher risk of all-cause mortality.

## 1. Introduction

Implantable cardioverter-defibrillators (ICDs) have become the primary method for preventing sudden cardiac death (SCD) in patients with heart failure with reduced left ventricular ejection fraction (HFrEF). However, the benefits of ICD implantation for primary SCD prevention have been shown to be inconsistent across the HFrEF population. Most patients with HFrEF who receive an ICD for primary SCD prevention will never experience ventricular arrhythmias or require ICD therapy (ATh), remaining at risk for adverse events associated with this therapy [1,2,3,4].

The utility of reduced left ventricular ejection fraction (LVEF) as a single parameter in predicting the benefit of prophylactic ICD implantation has been debated [3,4,5,6,7,8,9].

Therefore, predictors of ATh, in addition to LVEF, are being sought that would more accurately predict the benefit of ICD implantation for primary prevention of SCD. To this end, attempts are being made to identify patients at highest risk for developing life-threatening ventricular arrhythmias by identifying risk factors for ATh in patients with ICDs [10,11,12].

There are reports of an increased risk of ATh in patients with ICD implantation for primary SCD prevention and younger age, male sex, severe left ventricular systolic dysfunction (LVEF < 25%), non-sustained ventricular tachycardia, or the presence of chronic coronary artery occlusion (CTO) [10,11,12].

A risk prediction model (risk of VT/VF in a given patient compared with the risk of death from non-arrhythmic causes) was also developed among all patients with ICD implantation for primary prevention of SCD [13].

However, data in the literature are inconsistent. Risk factors for ATh are often sought based on the analysis of heterogeneous groups of patients with ischemic and non-ischemic HFrEF etiologies, and these groups differ in their risk of ventricular arrhythmias and SCD. Furthermore, a differential risk of arrhythmia and SCD is also observed in patients with ischemic HFrEF [2,14].

The literature is sparse and controversial regarding the prognostic significance of appropriate therapy.

Due to these controversies, we attempted to identify risk factors (predictors) for the development of ventricular arrhythmia in a homogeneous etiology (HFrEF) group of patients with ischemic heart disease and an ICD implanted for primary SCD prevention. In identifying risk factors, we considered clinical and echocardiographic parameters, parameters related to the severity of coronary artery disease, the method of myocardial revascularization, and the degree of ischemic left ventricular damage.

In addition, we attempted to create a proprietary model to estimate the probability of ventricular arrhythmia in the study group, based on the independent risk factors for ventricular arrhythmia identified. This model would allow the definition of a group of patients at the highest risk of ventricular arrhythmia.

### 1.1. Primary Objective

- Determination of risk factors for first appropriate therapy (ATh) among a number of clinical parameters (age, sex, comorbidities), electrocardiographic and echocardiographic parameters, the stage of coronary artery disease (presence of multivessel disease, presence of chronically occluded coronary arteries—CTO), the method of heart revascularization, pharmacotherapy, and the type of implanted device.
- Creation of a multiparameter logit model for estimating the probability of ATh occurrence, which will take into account independent risk factors for ATh.
- Determination of threshold values (based on ROC curve analysis) for risk factors for ATh.

### 1.2. Secondary Objective

- Survival time was assessed depending on the time that elapsed since the appropriate therapy.

### 1.3. Inclusion and Exclusion Criteria

#### 1.3.1. Inclusion Criteria

(a) Post-myocardial infarction or coronary artery disease with ischemic cardiomyopathy with LVEF < 35%.
(b) Implantation of an ICD or an ICD with resynchronization therapy (CRT-D) for the primary prevention of sudden cardiac death (SCD).
(c) Completed cardiac revascularization at least 3 months prior to ICD implantation.
(d) Previous myocardial infarction at least 3 months before the ICD implantation procedure.

#### 1.3.2. Exclusion Criteria

(a) Indications for ICD or CRT-D implantation for secondary prevention of SCD.
(b) Indication for ICD or CRT-D implantation for primary prevention of SCD for reasons other than ischemic myocardial damage with LVEF ≤ 35%.

## 2. Methodology

This paper is a retrospective analysis of a group of 260 patients hospitalized in the Cardiology Department of the Tadeusz Marciniak Lower Silesian Specialist Hospital in Wrocław during the years 2006–2019.

**Figure 1:**
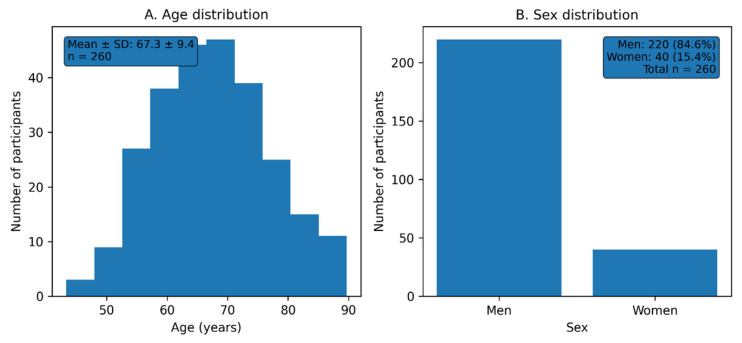
General characteristics of the study population. (**A**) Age distribution of the study population (mean ± SD). (**B**) Sex distribution of the study population.

The mean age was 67.3 ± 9.4 years. There were 40 women, constituting 15.4% of the group (Figure 1).

All patients were qualified for ICD or ICD with resynchronization function (CRT-D) implantation for the primary prevention of sudden cardiac death (SCD) on account of heart failure with reduced ejection fraction ≤ 35% progressing to ischemic heart damage. Post-infarction heart injury was diagnosed in 234 patients (90% of the group). Single-chamber cardioverter (ICD-VR) implantation was performed in 179 patients, dual-chamber cardioverter (ICD-DR) implantation in 61 patients, and cardioverter with resynchronization function (CRT-D) implantation in 20 patients, constituting 68.8%, 23.5%, and 7.7% of the group, respectively.

Follow-up was from ICD implantation until death or the last visit to the Implantable Device Care/Cardiology Clinic. The mean follow-up time was 4.47 ± 3.02 years.

Information on arrhythmic events was obtained from the documentation of the Implantable Device Monitoring Clinic. ICD follow-up and analysis of arrhythmic events were performed in each case by an electrophysiologist.

Due to the long observation period (2005–2019), there was no uniform standardization of ICD settings. Arrhythmia detection programming was performed in accordance with the official recommendations of implantable device manufacturers that were in effect at the time of implantation. In all devices, the ventricular tachycardia (VT) detection zone was programmed in the range of 172–188 beats per minute, with a number of intervals to detect (NID) from 30 to 40, and the ventricular fibrillation (VF) zone was set at ≥188 beats per minute, with NID in the range of 16–20/22. In the VT zone, arrhythmia discriminators (most commonly stability and morphology) were always programmed. In the VT zone, antitachycardia pacing (ATP, BURST type) was always used as the first therapy, delivered in sequences of 8–10 pulses at a cycle length equal to 88% of the VT cycle length; subsequent therapies were programmed as high-energy shocks at the maximum available energy (35–40 J). In the VF zone, high-energy shock therapy was programmed with one ATP sequence during ICD capacitor charging. The first appropriate therapy (ATh) was analyzed. Appropriate therapy (ATh) was defined as the first high-energy shock (CV) or antitachycardia pacing (ATP) triggered by a ventricular arrhythmia (ventricular tachycardia or ventricular fibrillation). The ventricular arrhythmia leading to ATh was classified as VT or VF based on ICD memory recordings, taking into account arrhythmia rate and the morphology of intracardiac ICD signals.

The ATh group included 79 patients (30.4% of the study group). Seventeen patients (6.5% of the study group) experienced electrical storms (≥3 ATh in 24 h).

### 2.1. Research Methods

Based on electrocardiographic (ECG) recordings from the last hospitalization before ICD implantation, the following parameters were analyzed: resting heart rate, presence of left bundle branch block (LBBB), presence of right bundle branch block (RBBB), and presence of QRS widening > 120 ms. Holter ECG recordings provided information on atrial arrhythmias and non-sustained ventricular tachycardias (nsVTs). Only arrhythmias that occurred before ICD implantation were included.

The analyzed echocardiographic studies were performed no earlier than 4 weeks before ICD implantation. All studies were conducted in accordance with the European Association of Cardiovascular Imaging (EACVI) guidelines (including Recommendations for Cardiac Chamber Quantification by Echocardiography in Adults: An Update from the American Society of Echocardiography and the European Association of Cardiovascular Imaging, 2015 [15]. The following echocardiographic parameters were used for further analysis: left ventricular ejection fraction (LVEF) assessed by the bi-plane Simpson disk method using the apical 4- and 2-chamber view, cardiac chamber dimensions, left ventricular end-diastolic dimension (LVEDd), left atrial anteroposterior dimension, right ventricular dimension, presence of a true left ventricular aneurysm, presence of severe, secondary, ischemic mitral regurgitation, and echocardiographic probability of pulmonary hypertension. The extent of regional left ventricular contractility abnormalities was determined based on the number of left ventricular akinetic segments (LAS). The classical division of the left ventricular myocardium into 17 segments was adopted. The segment was defined as akinetic when no systolic thickening was observed [15]. Each echocardiographic examination was interpreted by two experienced, accredited physicians.

### 2.2. Parameters of Coronary Heart Disease

Data regarding previous ST-segment elevation (STEMI) or non-ST-segment elevation (NSTEMI) myocardial infarctions were obtained from medical records. In all 260 patients, the last coronary angiography performed before ICD implantation was analyzed (up to 12 months before device implantation).

Chronic occlusion (CTO) of at least one of the three major arteries (LAD, Cx, or RCA) was defined as 100% occlusion of a coronary artery for a duration of greater than or equal to 3 months based on angiographic evidence.

Multi-vessel coronary artery disease (MVD) was classified as significant plaque buildup that narrows two or more of the three major coronary arteries (LAD, Cx, RCA) that supply blood to the heart.

The frequency of drug use was determined based on the information card (doctor’s recommendations for the patient) from the hospitalization during which the ICD implantation procedure was performed.

### 2.3. Statistical Analysis

Quantitative variables were characterized using the mean (M ± SD) or median and quartiles (Me [Q1, Q3]), depending on their adherence to the normal distribution (assessed by the Kolmogorov–Smirnov test with Lilliefors correction and the Shapiro–Wilk test). Qualitative variables were described by counts (*n*) and percentages (%). Differences in mean values between two groups were tested using Student’s *t*-test (after verifying variance homogeneity with Levene’s test) or the Mann–Whitney U test. Associations between categorical variables were tested using Pearson’s chi-square test. For dichotomous variables, Odds Ratios (OR) with 95% confidence intervals (CI) were reported.

Further statistical exploration involved several models and supplementary analyses. ROC analysis was conducted to establish optimal cut-off values for event predictors using Youden’s statistic; the Area Under the Curve (AUC), sensitivity, and specificity were calculated. A logistic regression model was subsequently developed to estimate the probability of appropriate therapy (Pr{ATh}) based on identified independent risk factors, with its threshold defined by the ROC curve. To assess the model’s discriminative ability, the AUC-ROC with a 95% CI was calculated. Model goodness-of-fit was evaluated using the Hosmer–Lemeshow test, while clinical utility was determined through decision curve analysis (DCA).

Decision curve analysis (DCA) was performed using R software (version 4.4.4; R Foundation for Statistical Computing, Vienna, Austria) with the dcurves package (version 0.4.0). Net benefit calculations were conducted using the dca() function. Overall survival (OS) was assessed using a Cox proportional hazards model with appropriate therapy (ATh) treated as a time-dependent covariate. All other statistical calculations were performed using Statistica PL v. 13.3 software. For all analyses, statistical significance was set at *p* < 0.05.

## 3. Results

First ATh occurred in 79 of 260 patients, which constituted 30.4% of the study group, including 17 patients (6.5%) who experienced electrical storm (ES), which was defined as three or more ATh within 24 h.

The time to the first ATh from ICD implantation was, on average, 21.1 ± 19.06 months (minimum 0.4, maximum 72 months). ATh occurred most frequently in the first and second year of follow-up (from ICD implantation).

**Figure 2:**
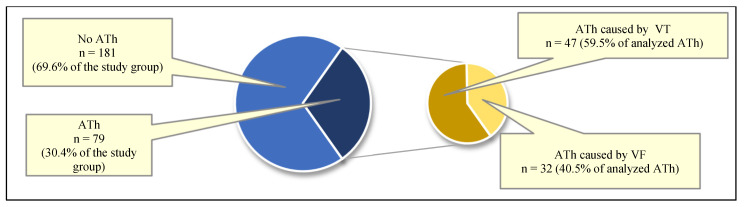
Structure of patients depending on the occurrence of appropriate therapy (ATh) (*n* = 79) and ventricular arrhythmia leading to ATh in the study group (*n* = 260).

In most cases, the cause of ATh was ventricular tachycardia (VT; 59.5% of ATh). Ventricular fibrillation (VF) was responsible for the remaining 40.5% of ATh. The cycle of ventricular tachycardias leading to ATh ranged from 340 ms to 260 ms, corresponding to a rate of 176 to 230/min (Figure 2; Table 1).

Appropriate therapy in response to ventricular arrhythmias (VT or VF) was associated with the delivery of therapy by ICD—low-energy therapy (ATP) in 35 ATh cases (44.3%), high-energy therapy (CV) in 33 ATh cases (41.8%), and ATP and CV in 11 ATh cases (13.9%).

During the 5-year follow-up period, 122 patients died, and all causes of death were analyzed.

Comparison of patients who experienced appropriate therapy (ATh) (*n* = 79) with the group of patients without appropriate therapy (*n* = 181).

The mean age of patients in the ATh group was lower compared to the non-ATh group (66.2 ± 8.6 years vs. 67.8 ± 8.6 years); however, no statistically significant difference in age was observed between the groups, either in terms of mean age or the age threshold of 64 years determined based on the ROC curve (*p* > 0.05).

Appropriate therapy (ATh) was observed in 13 women and 66 men, representing 16.5% and 83.5% of the ATh group, respectively. No statistically significant sex differences were observed in relation to the occurrence of ATh (*p* > 0.05).

### 3.1. NYHA Functional Class and Comorbidities

No significant differences were observed between the ATh and non-ATh groups in terms of heart failure symptoms according to the NYHA classification.

### 3.2. Electrocardiographic Parameters and Arrhythmias

In the ATh group, non-sustained ventricular tachycardias (nsVT) occurring before ICD implantation were significantly more frequent: 46 patients (58.2%) vs. 64 patients (35.4%) in the non-ATh group (*p* < 0.001).

There were no significant differences between the ATh and non-ATh groups in the incidence of paroxysmal and permanent atrial fibrillation.

### 3.3. Echocardiographic Parameters

In the ATh group, the mean left ventricular ejection fraction (LVEF) was 29.4% (SD ± 5.0) vs. 30.0% (SD ± 4.9) in the non-ATh group. There were no statistically significant differences between the groups in terms of mean LVEF or when the cut-off points determined based on the ROC curve for LVEF were 28% (*p* > 0.05).

Based on the ROC curve, a threshold value for LVEDd was determined that optimally differentiated the groups. The proposed cut-off point for LVEDd was 68 mm.

In the ATh group, LVDd ≥ 68 mm was observed in 48 (60.7%) patients vs. 63 (34.8%) patients in the non-ATh group. This difference was statistically significant (*p* < 0.001).

The extent of regional left ventricular contractility abnormalities was determined based on the number of left ventricular akinetic segments (LAS). The classical division of the left ventricular myocardium into 17 segments was adopted.

Based on the ROC curve for estimating the probability of ATh based on LAS, a threshold value of 7 was proposed. LAS ≥ 7 was found in 45 patients (56.9%) of the ATh group and in 70 patients (38.6%) of the non-ATh group. The difference was statistically significant (*p* = 0.007).

No significant differences were found between the groups with and without ATh for the following echocardiographic parameters: presence of left ventricular aneurysm, left atrial diameter, right ventricular diameter, presence of significant mitral valve regurgitation, and high probability of pulmonary hypertension estimated by echocardiography (*p* > 0.05).

### 3.4. Coronary Heart Disease (Angiographic Findings, Revascularization)

Previous myocardial infarction (at least 3 months before ICD implantation) was diagnosed in 62 (91.1%) patients in the ATh group vs. 162 patients (89.5%) in the non-ATh group (statistically insignificant difference, *p* = 0.875).

Coronary angiography performed up to 12 months before ICD implantation was analyzed. Chronic occlusion (CTO) of at least one of the three major arteries (LAD, Cx, or RCA) was detected in 45 (57%) patients in the ATh group vs. 78 (43.1%) patients in the non-ATh group. The difference was statistically significant (*p* = 0.039).

Multi-vessel coronary artery disease (MVD) involving ≥2 major vessels (LAD, Cx, RCA) was found significantly more frequently in the ATh group: 43 patients (54.4%) vs. 74 patients (40.9%) in the non-ATh group (*p* = 0.045).

Cardiac revascularization (completed at least 3 months before ICD implantation) was performed in 72 patients in the ATh group (91.1%) vs. 155 patients in the non-ATh group (85.6%). Seven patients in the ATh group (8.9%) and twenty-six patients in the non-ATh group (14.4%) did not undergo myocardial revascularization due to lack of patient consent or lack of expected benefit. The differences were not statistically significant (*p* = 0.306).

Percutaneous coronary interventions (PCI) were performed non-significantly more often in the non-ATh group: 131 patients (72.4%) vs. 51 patients (64.5%) in the ATh group (*p* = 0.205).

Coronary artery bypass grafting (CABG) was performed significantly more frequently in the ATh group: 34 patients (43%) vs. 43 patients (23.7%) in the non-ATh group (*p* = 0.001). The mean time from CABG to ICD implantation was 8.9 ± 6.3 years. In the ATh group, patients with time ≥ 10 years from CABG to ICD implantation constituted 17.7% (*n* = 14) vs. 10.4% (*n* = 19) in the non-ATh group.

No statistically significant difference was observed when comparing the ATh group with the non-ATh group in terms of the type of implanted ICD-VR and ICD-DR devices (*p* > 0.05). ICD with cardiac resynchronization function (CRTD) was more frequently implanted in the ATh group (12.7% vs. 5.5% in the non-ATh group); however, the differences were not statistically significant (*p* = 0.083).

### 3.5. Pharmacotherapy

The frequency of medication use was determined based on the patient information sheet (patient recommendations) from the hospitalization during which the ICD was implanted. No significant differences were found between patients with and without ATh in the frequency of use of the main drug classes (*p* > 0.05). Both groups had high rates of use of beta-blockers (ACEI/ARB) and statins. Amiodarone was more frequently prescribed in the ATh group (22.8% vs. 15.5% in the group without ICD), but this difference was not significant (*p* = 0.155).

### 3.6. Logistic Regression Analysis of the Probability of Appropriate ICD Therapy (ATh)

To assess the impact of the analyzed clinical parameters on the risk of ATh in the 260 study patients, univariate and multivariate logistic regression analyses were used. The results of the analyses are presented in Table 2.

### 3.7. Univariate Logistic Regression Analysis

Univariate logistic regression analysis showed that the strongest risk factor for ATh in the study group of patients (*n* = 260) was left ventricular end-diastolic diameter (LVEDd) ≥ 68 mm, with odds ratio (OR) = 2.75 (i.e., the risk of ATh for patients with LVEDd ≥ 68 mm was 2.75 times higher compared to patients with LVEDd < 68 mm).

Other parameters associated with an increased risk of ATh include the following:

1. Previous coronary artery bypass grafting (CABG) (OR = 2.66)
2. Non-sustained ventricular tachycardia (nsVT) occurring before ICD implantation (OR = 2.47)
3. Number of akinetic left ventricular segments (LAS) ≥ 7 (left ventricular contractility disorders, assessed by echocardiography) (OR = 2.1)
4. Presence of chronic occlusion (CTO) in one of the main coronary arteries (LAD, Cx, or RCA) (OR = 1.75)
5. Multivessel coronary artery disease involving ≥2 major vessels, i.e., LAD, Cx, or RCA (revascularization of ≥2 major vessels or presence of CTO in ≥1 of the major vessels and revascularization of ≥1 other major vessel) (OR = 1.73)

### 3.8. Multivariate Logistic Regression Analysis

Multivariate logistic regression analysis was performed to determine independent risk factors for ATh. The results are presented in Table 2.

The independent risk factors for ATh in the studied group of patients (*n* = 260) were as follows (starting with the highest odds ratio—OR):

1. Previous coronary artery bypass grafting (CABG) (OR = 3.25)
2. Left ventricular end-diastolic dimension (LVEDd) ≥ 68 mm (OR = 2.95)
3. Non-sustained ventricular tachycardia (nsVT) diagnosed before ICD implantation (OR = 2.75)
4. Number of akinetic left ventricular segments ≥ 7, assessed on the basis of echocardiography (OR = 2.69)

### 3.9. Logit Model for Estimating the Probability of an Appropriate ICD Therapy—Pr {ATh}

Based on the independent risk factors (predictors) of ATh occurrence in the studied group of patients, a logit model was created, i.e., an expression allowing estimation of the probability of occurrence of an appropriate therapy Pr {ATh}, which takes the logit form

*Pr* (*ATh* = 1|*X*) = −2.674 + 1.081 × (*LVDd* ≥ 68 mm) + 1.178 × (*CABG*) + 1.012 × (*nsVT*) + 0.988 × (*LAS* ≥ 7)

The risk factors taken into account in the expression take the value 1 (a given factor is present) or 0 (a given factor is not present).

**Figure 3:**
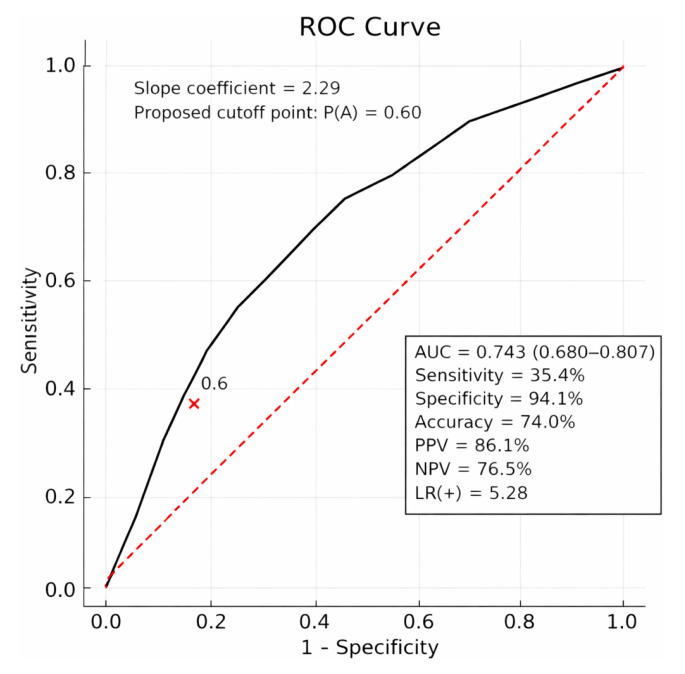
ROC curve of the logistic model as a predictor of the probability of appropriate ICD therapy. Area under the curve (AUC), sensitivity, specificity, accuracy, positive predictive value (PPV), negative predictive value (NPV), and likelihood ratio for a positive test result (LR+) for a cut-off value of Pr {ATh = 1|X} ≥ 0.60.

The proposed cut-off point for the logit model was 0.60, and the classification quality of the model was determined based on the ROC curve and is presented in Figure 3.

The model analysis for the study group showed that the risk of ATh is more than six times higher among patients whose probability estimated based on the logit model is Pr {ATh} ≥ 0.6 compared to patients with Pr {ATh} < 0.6 (OR = 6.08) (Table 3).

To facilitate the use of the logit model, a Pr {ATh} calculator was developed. Example Pr {ATh} results calculated using the calculator are presented in Supplementary Materials. The proposed cut-off point for the logit model was 0.60. (A) positive result Pr {ATh} ≥ 0.60, (B) negative result Pr {ATh} < 0.60.

**Figure 4:**
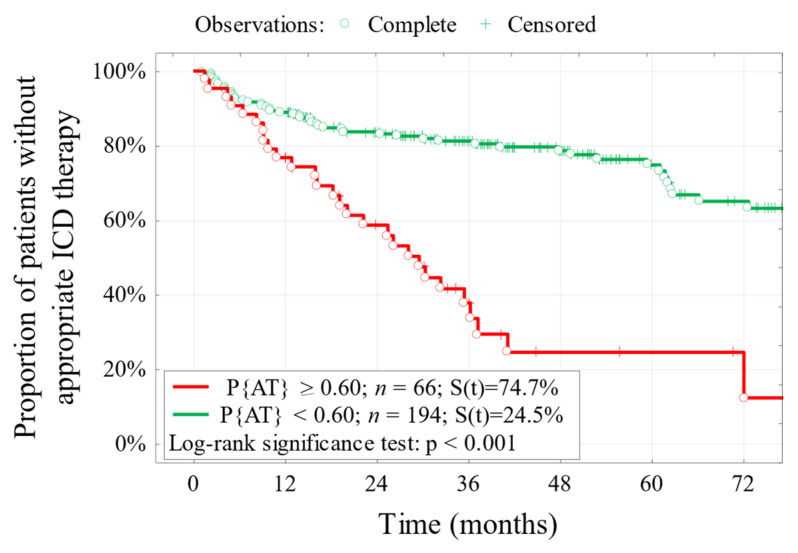
Curves of ICD-free survival (ATh) in groups of patients differing in the probability level estimated using the logit model P {ATh} ≥ 0.60. The result of the *p* significance test and the five-year probability of ATh-free survival. *n*—number of patients, Y-axis—percentage of patients without ATh experience, X-axis—follow-up time. Pr {ATh}—probability of ATh occurrence based on the logit model.

The probability of 5-year ATh-free survival (the percentage of patients without ATh after 5 years of follow-up) was lower in the group of patients with the logit model result Pr {ATh} ≥ 0.60 compared to Pr {ATh} < 0.60 and was 21.7% vs. 77.4% (Figure 4).

The logistic regression model demonstrated high performance and validity. The model’s discriminatory ability was high, as evidenced by ROC analysis. Calibration was confirmed by the Hosmer–Lemeshow test (χ^2^ = 4.90, *p* = 0.557), which indicated no significant difference between the predicted and observed outcomes. Furthermore, decision curve analysis (DCA) demonstrated that the model maintained a superior net benefit compared with the “treat all” and “treat none” strategies over a threshold probability range of 5–50%. (A figure showing the decision curve analysis of the ATh predictive model is included in the Supplementary Materials.)

The logit model presented above only allows for the prediction of an increased risk of appropriate therapy in patients with an ICD implanted for primary prevention of SCD (HFrEF of ischemic etiology).

### 3.10. Overall Survival Analysis for the Study Group

**Figure 5:**
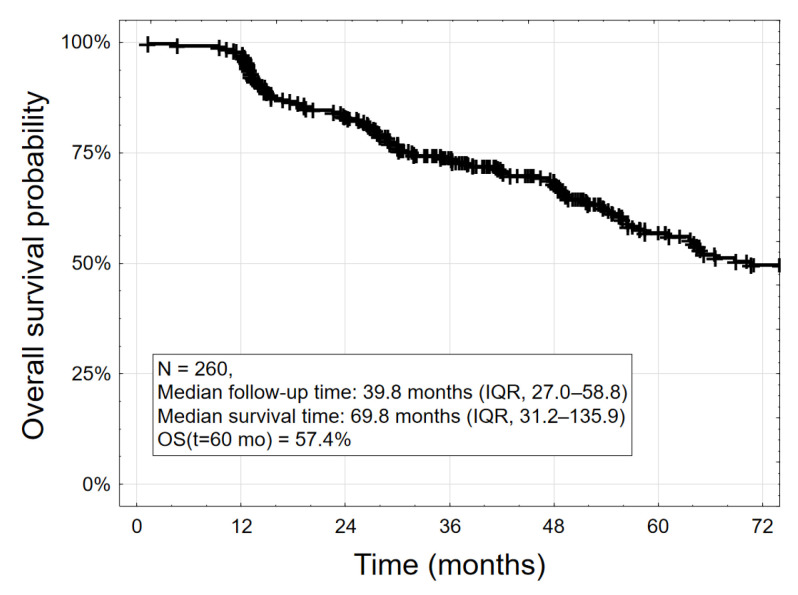
Kaplan–Meier curve of overall survival for 260 patients from the date of device implantation.

The median survival for the entire study group (*n* = 260) was 69.8 months, meaning that 50% of the patients would die within 5.9 years. The five-year survival probability S (t = 60 months) was 57.4% (Figure 5). The median follow-up time was 39.8 months (IQR: 27.0–58.8 months).

### 3.11. Overall Survival Analysis for the Study Group Depending on the Occurrence of Appropriate ICD Therapy (ATh)

Using a Cox proportional hazards model with appropriate therapy (ATh) as a time-dependent variable, we demonstrated that ATh was linked to a significantly increased risk of mortality (HR = 1.69; 95% CI: 1.15–2.48; *p* = 0.008). These findings indicate that ICD intervention serves as a robust predictor of poor clinical outcomes in these patients.

**Figure 6:**
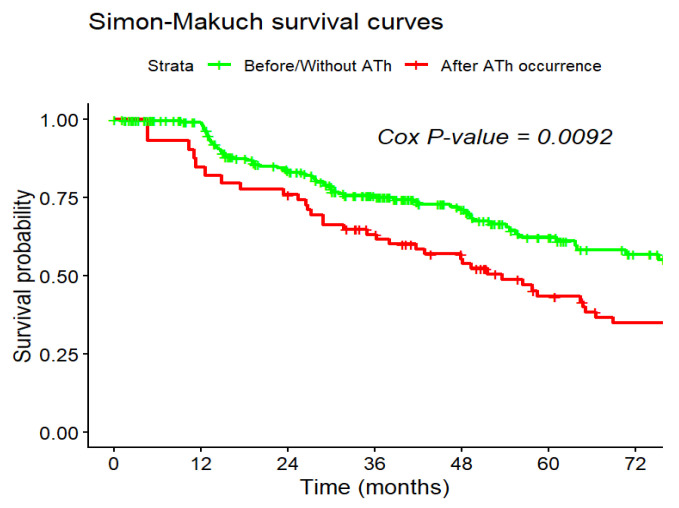
Simon-Makuch survival plot showing the effect of ATh occurrence. Analysis performed with ATh treated as a time-dependent covariate. Cox *p*-value = 0.009.

The Simon-Makuch survival curves demonstrated a clear divergence in survival probability following the occurrence of an appropriate therapy (ATh). At 60 months (5 years) post-implantation, the estimated survival probability was approximately 61% for the period before/without ATh, compared to 43% after the occurrence of ATh. The median survival time was substantially shorter in the post-therapy group (approximately 56 months) than in the group without intervention (approximately 102 months) (Figure 6).

## 4. Discussion

Patients with heart failure and reduced left ventricular ejection fraction (HFrEF) of ischemic etiology are at high risk of sudden cardiac death (SCD). The benefits of ICD implantation in terms of reduced mortality are not uniform in this patient population and come at a high cost: Appropriate ICD therapy, which adversely affects quality of life (QoL), increases the risk of heart failure progression, and increases the risk of death [16,17]. Therefore, it is justified to identify risk factors for inappropriate therapy and define the group of patients particularly at risk for their occurrence.

In the MADIT II study, appropriate therapy was observed in 24% of patients with ICD (mean follow-up was 21 months) [16]. Interestingly, data from observational studies show a lower incidence of ATh compared to randomized trials [18]. In our study, ATh occurred in 79 of 260 patients, representing 30.4% of the study group. The higher percentage of patients with ATh compared to the literature data can be explained by the longer follow-up time (mean follow-up in our study was 4.47 ± 3.02 years).

### 4.1. The Influence of Selected Demographic and Clinical Factors on the Occurrence of ATh

The effect of age on the risk of ATh is not clear. The benefits of ICD implantation for primary prevention of SCD are lower in older patients compared to younger patients, which may be explained by the increasing risk of death from non-arrhythmic causes with age [13,19]. In our study, we did not demonstrate a significant association between age and the risk of ATh.

A meta-analysis by Conen et al. (patients with ICD implanted for primary prevention of SCD) demonstrated that women had a significantly lower risk of ATh compared to men [20]. Similarly, Frodi DM et al., in a study of 2998 patients (primary and secondary prevention), demonstrated that female sex was associated with a lower risk of ATh [21]. In our study, we did not demonstrate a relationship between sex and the risk of ATh. This may be due to the limited number of women in the group (15.3%).

NYHA functional class III has been shown to be an independent risk factor for death in patients with HFrEF and an ICD implanted for primary prevention of SCD [22]. The relationship between NYHA functional class and the risk of ATh remains a matter of debate. The literature provides evidence of lower benefits from prophylactic ICD implantation in patients with HFrEF in NYHA functional class III compared to patients in NYHA functional class II [23]. Zeitler et al., based on a meta-analysis of the MADIT II and SCD-HeFT studies, demonstrated that the risk of ATh was higher in patients with NYHA functional class II and III compared to asymptomatic patients (NYHA functional class I) [12]. Van der Lingen ACJ et al. [6] demonstrated that ATh was more common in class I compared to class II–III, which was consistent with the registry study by Sabbag Avi et al., who demonstrated that asymptomatic patients had twice as many ATh events as symptomatic patients. It has been suggested that asymptomatic patients are prone to exercise-induced arrhythmia [24]. In our study, we found no relationship between NYHA functional class and the risk of ATh.

### 4.2. The Influence of Selected Electrocardiographic Parameters and Arrhythmias on the Occurrence of ATh

Among electrocardiographic parameters and arrhythmias as potential predictors of ATh, resting heart rate (HR), QRS width, and non-sustained ventricular tachycardias (nsVT) are of particular interest.

In our study, we did not demonstrate an association between resting heart rate and the risk of ATh, which is consistent with the sub-analysis of the MADIT II study, where no association was found between resting heart rate ≥ 80/min and the risk of ATh [25].

Different results were presented based on the meta-analysis by Younis et al., which showed a correlation between resting heart rate > 75/min and an increased risk of ventricular arrhythmia defined as VT > 200/min or VF [13].

Data on the relationship between QRS complex widening and the risk of ATh are debated. Maciąg et al. demonstrated an association between QRS complex widening ≥ 140 ms and an increased risk of ATh in a group of patients with ischemic heart disease and ICD implanted for primary prevention [26]. In the present study, we found no relationship between QRS complex width > 120 ms, the presence of right or left bundle branch block, and the risk of ATh, which is consistent with the results of Singh et al. [25].

Non-sustained ventricular tachycardia (nsVT) has attracted attention for many years as a marker of increased risk of ventricular arrhythmias and sudden cardiac death. In our study, non-sustained ventricular tachycardia (nsVT) detected before ICD implantation was found to be an independent risk factor for ATh, which is consistent with the literature data [13,27,28].

### 4.3. The Influence of Selected Echocardiographic Parameters on the Occurrence of ATh

The influence of EF on the incidence of ATh in the population of patients with ischemic etiology is not clear and remains a subject of debate. In our study, LVEF was not found to be a risk factor for ATh, which is consistent with the results of Singh et al. [25], who, based on the analysis of the MADIT II study, showed no association between LVEF and the incidence of ATh, but is in contrast to the results of a meta-analysis of the MADIT-II, MADIT-CRT, MADIT-RIT, and MADIT-RISK studies, which showed that LVEF ≤ 25% was an independent risk factor for VT > 200/min or VF [13].

In recent years, there has been increasing criticism of impaired ejection fraction as a criterion for ICD implantation in patients after myocardial infarction. Clinical trials and registries are being conducted to select patients at low risk of SCD among those with severe EF impairment and those at high risk of SCD among those with moderately reduced or preserved EF [29]. Therefore, further research is required for a possible revision of the current recommendation.

Data regarding the association of end-diastolic volume (LVEDd) with the risk of ATh are limited. However, left ventricular dilation has been implicated in an increased risk of ATh. Aleong et al., based on a sub-analysis of the GRADE study of 930 patients with ICD, 74% of whom had ICD implanted for primary prevention, demonstrated that the risk of ATh increased by 33% for each 10 mm increase in LVEDd [30]. Our study demonstrated that LVEDd was significantly greater in the ATh group. An LVEDd of ≥68 mm, determined based on the ROC curve, was found to be an independent risk factor for ATh. Left ventricular dilation, as a consequence of unfavorable left ventricular remodeling, is believed to increase the risk of ventricular arrhythmia due to impaired cellular ion channel function and prolonged myocyte action potential [31].

The relationship between regional left ventricular contractility disorders and the risk of death and ventricular arrhythmias has been the subject of research for many years.

Modern imaging methods such as magnetic resonance imaging (MRI) allow for precise assessment of the post-infarction scar area, which may be a substrate for ventricular reentry arrhythmia [32]. A study by Wu et al. demonstrated that the size of the left ventricular myocardial scar assessed by cardiac MRI correlates with an increased risk of ATh in patients with prophylactic ICD implantation [33]. However, MRI has limited use in everyday clinical practice. Mahenthiran et al. demonstrated that, based on echocardiography, a Wall Motion Score Index (WMSI) ≥ 1.5 and the number of akinetic left ventricular segments ≥ 2 within the right coronary artery vascularization were risk factors for the composite endpoint (ATh or death) [34]. In our study, we demonstrated that the number of akinetic left ventricular segments (LAS) ≥ 7 was an independent risk factor for ATh.

### 4.4. The Influence of the Degree of Coronary Artery Disease and the Method of Revascularization on the Occurrence of ATh

Multivessel coronary artery disease is associated with an increased risk of cardiovascular events and death in patients with a history of myocardial infarction [35]. However, the impact of multivessel coronary artery disease on the risk of ATh is not clear.

Chronic coronary artery occlusion (CTO) is found in 30–70% of patients with ischemic heart damage and HFrEF. CTO has been associated with an increased risk of death and ventricular arrhythmias in the HFrEF population [36,37].

In the study by van Dongen et al. (the eCTOpy-in-ICD Study, patients with ICD implanted for primary prevention (74%) and secondary prevention SCD), CTO was shown to be an independent risk factor for ATh [38]. A similar sub-analysis of the VACTO study showed that CTO in the major coronary arteries was an independent risk factor for ATh [32].

In our study, chronic occlusion (CTO) of at least one of the three major coronary arteries was found in 47.3% of patients, significantly more often in patients who experienced ATh, and the presence of CTO was shown to be a risk factor for ATh, although only in univariate analysis. The impact of CTO opening procedures on the risk of ATh was not analyzed.

In patients with post-infarction heart damage and ICD, the risk of ventricular arrhythmia remains high despite revascularization. This is consistent with literature data, as there are reports of an increased risk of ventricular arrhythmia with increasing time since revascularization [39,40].

In our study, patients who experienced ATh were significantly more likely to undergo coronary artery bypass grafting (CABG), which was found to be an independent risk factor for ATh. Furthermore, the mean time from CABG to ICD implantation was 8.9 ± 6.3 years, and patients ≥ 10 years from CABG to ICD implantation constituted 17.7% of the ATh group vs. 10.4% of the non-ATh group. A sub-analysis of the MADIT-CRT study (759 patients with post-infarction heart failure and ICD implanted for primary prevention, 612 patients with prior revascularization, 3-year follow-up) showed that the risk of ventricular arrhythmia (VT or VF) and the risk of ATh increased with each additional year between revascularization and ICD implantation. The beneficial effect of revascularization on the risk of ventricular arrhythmias and SCD appears to decrease over time [40].

Based on the independent risk factors for ATh identified in the study group, we developed our own multiparameter logistic model that estimates the probability of ATh occurrence (Pr {ATh}). The proposed model predicts an increased risk of receiving appropriate ICD therapy for ventricular arrhythmia; however, it is not intended to estimate the overall clinical benefit of ICD implantation, as it does not account for non-arrhythmic mortality. The calculator should be used with caution, as external validation confirming its generalizability and reliability beyond the studied patient cohort has not been performed.

### 4.5. Impact of Appropriate ICD Therapy on Survival

Data from randomized trials indicate an increased risk of death associated with ATh. A sub-analysis of the SCD-HeFT trial (patients with HFrEF and an ICD implanted for primary prevention of SCD) showed a more than 5-fold increase in the risk of death in the group of patients who experienced ATh compared to patients without ATh [41].

Similar conclusions were obtained from the MADIT II study—a population of patients after myocardial infarction, with LVEF ≤ 30%, ICD implanted for primary prevention, where ATh (CV or ATP) was associated with significantly higher all-cause mortality (over 3.4X) [16].

We obtained similar results in our study—appropriate therapy was associated with a statistically significant reduction in survival time.

### 4.6. Gaps in Evidence and Future Directions

Speckle-tracking echocardiography, including regional strain, global longitudinal strain (GLS), and myocardial dispersion (MD), has been shown to be associated with an increased risk of VAs, whereas strain measures of paradoxical motion and myocardial work still need to be explored further in this context. GLS and MD have been most widely investigated and may provide important information for assessing the risk of VAs in several settings, even in patients with LVEF > 35%. Consequently, they could be useful for identifying patients who could stand to benefit from an ICD for the prevention of sudden cardiac death [42,43,44].

The MADIT-RIT study demonstrated that ICD programming strategies based on higher heart rate thresholds or delayed therapy significantly reduced the risk of therapies (inappropriate and those resulting from transient ventricular arrhythmias) and were associated with a significant reduction in all-cause mortality without increasing the risk of sudden cardiac death. These results confirm that less aggressive ICD programming is a key modifiable factor influencing long-term outcomes in patients with primary prevention ICDs [45].

The current dynamic development of ablation techniques shows that early VT ablation—after the first appropriate ICD therapy in patients with ischemic and non-ischemic LV damage—improves survival (significant reduction of the composite endpoint: death or HF hospitalization)—PARTITA study [46].

The study by de Diego et al. aimed to evaluate the effect of ARNI on ventricular arrhythmias and appropriate ICD therapies directly in remotely monitored ICD/CRT-D patients, comparing periods of ACEi/ARB use with periods after initiation of ARNI use (population with chronic heart failure HFrEF, ejection fraction ≤ 40%, NYHA functional class ≥ II). A significant reduction in the frequency of ATh, a significant reduction in the number of PVCs per hour, and increased biventricular pacing were observed [47].

## 5. Limitations

A limitation of this study is that the model’s usefulness in estimating the probability of ATh was demonstrated for a group of 260 patients from a single center, which precludes its widespread application. This model is exploratory in nature and requires thorough internal and external validation, so its results should be interpreted with caution until confirmed in independent cohorts. The analysis considered all-cause mortality (arrhythmic and non-arrhythmic mortality were not analyzed separately). Only the time to first therapy was analyzed (relapses were not analyzed). The study is based on retrospective data analysis, which limits access to data and the ability to draw causal conclusions. Arrhythmic events during ICD monitoring were assessed by a single physician (electrophysiologist). ICD memory records were not reviewed by a second physician. ICD programming was not standardized across the study period. Changes in device technology, programming strategies, heart failure pharmacotherapy (sacubitril/valsartan and SGLT2 inhibitors), and VT ablation techniques over time may have influenced the observed incidence of ventricular arrhythmia and appropriate therapy.

These aspects should be considered as a reason for cautious generalization of the results to contemporary practice.

## 6. Conclusions

In our study, independent risk factors for ATh were found to include: the number of akinetic left ventricular segments ≥ 7, left ventricular end-diastolic diameter (LVEDd) ≥ 68 mm, non-sustained ventricular tachycardia (nsVT) observed before ICD implantation, and prior CABG surgery (in patients with long-standing and advanced coronary artery disease). Based on these independent risk factors, we developed our own multiparameter logit model to estimate the probability of developing ATh, which was higher in the group of patients with a logit model score Pr {ATh} ≥ 0.60 compared to any group of patients with a single ATh risk factor. This model should be interpreted with caution because it does not predict the overall clinical benefit of ICD implantation, but only the probability of appropriate intervention resulting from ventricular arrhythmia. The occurrence of ATh was associated with a significantly higher risk of all-cause mortality.

## Figures and Tables

**Table 1:** General characteristics of the study group (*n* = 260).

Feature (Variable)	Total *N* = 260
Echocardiographic parameters:	
Left ventricular ejection fraction EF (%):	
M ± SD	29.8 ± 4.9
Me [Q1; Q3]	30 [25; 35]
Min–Max	18–35
NYHA class:	
1, *n* (%)	40 (15.4%)
2–3, *n* (%)	218 (83.8%)
Comorbidities:	
Arterial hypertension	200 (76.9%)
Diabetes mellitus	93 (35.8%)
Nicotine use	90 (34.6%)
CKD (GFR < 60 mL/min/1.73 m^2^)	96 (35.8%)
Previous ischemic stroke	37 (14.2%)
Carotid artery atherosclerosis	26 (10.0%)
Peripheral artery disease	24 (9.2%)
COPD	32 (12.3%)
Arrhythmias before ICD implantation:	
Presence of nsVT	110 (42.3%)
Paroxysmal atrial fibrillation	39 (15.0%)
Persistent atrial fibrillation	32 (12.3%)
Coronary artery disease:	
Previous myocardial infarction, *n* (%)	234 (90.0%)
Multivessel coronary artery disease	117 (45.0%)
Presence of CTO in LAD or Cx or RCA	123 (47.3%)
Myocardial revascularization:	
PCI, *n* (%)	182 (70.0%)
CABG, *n* (%)	77 (29.6%)
PCI + CABG	32 (12.3%)
No revascularization	33 (13.8%)
Type of implanted device:	
ICD DR, *n* (%)	61 (23.5%)
ICD VR, *n* (%)	179 (68.8%)
CRT-D, *n* (%)	20 (7.7%)

Abbreviations: M—mean; SD—standard deviation; Me—median (50%); Q1—lower quartile (25%); Q3—upper quartile (75%); Min—minimum value; Max—maximum value; *n*—number; %—proportion. PCI—percutaneous coronary intervention; CABG—coronary artery bypass grafting; LAD—left anterior descending artery; Cx—circumflex artery; RCA—right coronary artery; CTO—chronic total occlusion; nsVT—non-sustained ventricular tachycardia; CKD—chronic kidney disease; COPD—chronic obstructive pulmonary disease; ICD DR—dual-chamber implantable cardioverter-defibrillator; ICD VR—single-chamber implantable cardioverter-defibrillator; CRT-D—cardiac resynchronization therapy defibrillator; NYHA—New York Heart Association.

**Table 2:** Results of logistic regression analyses of the probability of appropriate ICD therapy in the group of 260 patients.

Predictors of Appropriate ICD Therapy	Univariate Analysis B	Univariate Analysis *p*	Univariate Analysis OR (95% CI)	Multivariate Analysis B	Multivariate Analysis *p*	Multivariate Analysis OR (95% CI)
Age < 64 years	0.446	0.109	1.56 (0.91–2.70)	-	-	-
LVEF < 28%	0.307	0.301	1.36 (0.76–2.43)	-	-	-
Multivessel coronary artery disease	***0.546***	***0.045***	***1.73 (1.01–2.95)***	0.168	0.664	1.18 (0.55–2.54)
QRS width > 120 ms	−0.063	0.840	0.94 (0.51–1.73)	−0.008	0.819	0.92 (0.47–1.82)
LVDd ≥ 68 mm	***1.012***	***<0.001***	***2.75 (1.59–4.75)***	***1.081***	***<0.001***	***2.95 (1.63–5.32)***
Previous CABG	***0.980***	***0.001***	***2.66 (1.51–4.71)***	***1.178***	***<0.001***	***3.25 (1.73–6.10)***
Presence of nsVT before ICD implantation	***0.935***	***0.001***	***2.55 (1.48–4.39)***	***1.012***	***0.001***	***2.75 (1.52–4.97)***
Number of akinetic LV segments ≥ 7	***0.741***	***0.007***	***2.10 (1.22–3.60)***	***0.988***	***0.001***	***2.69 (1.47–4.91)***
Presence of CTO in LAD or Cx or RCA	***0.558***	***0.041***	***1.75 (1.02–2.99)***	0.504	0.093	1.66 (0.92–2.98)

B—model parameter estimate (regression coefficient); *p*—probability level of the significance test; OR—odds ratio for a unit change in the parameter; 95% CI—lower and upper bound of the 95% confidence interval for the odds ratio of CABG (coronary artery bypass grafting), LAD—left anterior interventricular branch of the coronary artery, Cx—circumflex branch, RCA—right coronary artery, CTO—chronic total occlusion, nsVT—non-sustained ventricular tachycardia, LVDd—left ventricular diastolic diameter. Multivessel coronary artery disease defined as: status after revascularization of ≥2 major vessels (LAD, Cx, RCA) or presence of CTO in ≥1 of the major vessels (LAD, Cx, RCA) and status after revascularization of ≥1 other major vessel (LAD, Cx, RCA).

**Table 3:** Logistic model analysis for the probability of appropriate therapy—Pr{ATh} in the study group of 260 patients.

Logistic Model	Appropriate Therapy		χ^2^ Test	OR (95% CI)
	Yes *N* = 79	No *N* = 181		
Pr {ATh} ≥ 0.60	*n* = 51 (64.6%)	*n* = 15 (8.3%)	***p* < 0.001**	**6.08 (3.01–12.3)**
Pr {ATh} < 0.60	*n* = 28 (35.4%)	*n* = 166 (91.7%)		1.00 (ref.)

*n*—number; %—proportion (structure index); OR—odds ratio; 95% CI—lower and upper bound of the 95% confidence interval for odds ratio; *p*—significance test.

## Data Availability

The data can be obtained by contacting the corresponding author.

## Supplementary Materials

The following supporting information can be downloaded at: https://www.mdpi.com/article/10.3390/jcm15031033/s1, File S1: Decision curve analysis of the ATh predictive model; File S2: Examples of calculating the probability of adequate intervention supplementary.
