# Supplementary material for: Predictors and Prognostic Significance of Appropriate Implantable Cardioverter-Defibrillator Therapy in Primary Prevention Patients with Ischemic Cardiomyopathy
Source: J Clin Med. 2026 Jan 28;15(3):1033. doi: 10.3390/jcm15031033 (PMC12898167; doi:10.3390/jcm15031033)
Supplement: Supplementary file 1 [file jcm-15-01033-s001.zip › File S1 Decision curve analysis of the ATh predictive model.pdf]

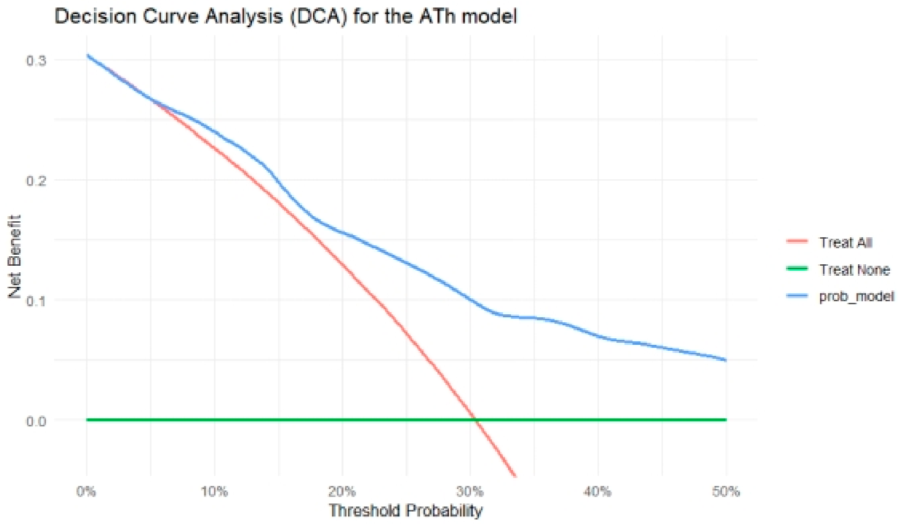

Figure S1. Decision curve analysis of the ATh predictive model. The blue line indicates the model's Net Benefit, which exceeds both 'Treat All' (red) and 'Treat None' (green) strategies across the 0–50% threshold probability range, demonstrating superior clinical utility
