# Supplementary material for: Predictors and Prognostic Significance of Appropriate Implantable Cardioverter-Defibrillator Therapy in Primary Prevention Patients with Ischemic Cardiomyopathy
Source: J Clin Med. 2026 Jan 28;15(3):1033. doi: 10.3390/jcm15031033 (PMC12898167; doi:10.3390/jcm15031033)
Supplement: Supplementary file 1 [file jcm-15-01033-s001.zip › File S2 Examples of calculating the probability of adequate intervention supplementary.pdf]

Examples of calculating the probability of appropriate intervention based on the Pr {ATh} calculator on the proposed logit model

Calculator for calculating the probability of an appropriate intervention Pr{ATh}

LVDd ≥ 68 mm = **1**; LVDd <68 mm = **0**	**1**
Number of akinetic segments≥ 7 = **1**; LAS <7 = **0**	**1**
Previous CABG Yes = **1**; No = **0**	**0**
Non-sustained ventricular tachycardia (nsVT): Yes = **1**; No = **0**	**1**
Probability of appropriate intervention : Pr{*ATh*} =	**0,600**
**High probability of ATh**	**YES**

Calculator for calculating the probability of an appropriate intervention Pr{ATh}

LVDd ≥ 68 mm = **1**; LVDd <68 mm = **0**	**0**
Number of akinetic segments ≥ 7 = **1**; LAS <7 = **0**	**0**
Previous CABG Yes = **1**; No = **0**	**0**
Non-sustained ventricular tachycardia (nsVT): Yes = **1**; No = **0**	**1**
Probability of appropriate intervention: Pr{*ATh*} =	**0,159**
**High probability of ATh**	**No**
